# Long-term Survival in a Patient with Recurrent Pulmonary Metastases from Uterine Leiomyosarcoma

**DOI:** 10.7759/cureus.405

**Published:** 2015-12-15

**Authors:** Gwenalyn Garcia, Shiksha Kedia, Meekoo Dhar

**Affiliations:** 1 Department of Hematology and Oncology, Staten Island University Hospital

**Keywords:** leiomyosarcoma

## Abstract

Chemotherapy is the standard of care for disseminated uterine leiomyosarcoma; however, long-term survival is rarely achieved with this aggressive disease. We report a patient with recurrent pulmonary metastatic disease from uterine leiomyosarcoma who is doing well without significant disease nine years after her initial diagnosis. We discuss her clinical course, the treatment modalities she received, and the clinical evidence supporting these.

## Introduction

Leiomyosarcoma of the uterus is an aggressive tumor with a median overall survival of two years in patients presenting with disseminated disease [1]. Treatment is surgery for resectable disease while results with chemotherapy and radiation have been suboptimal [2-6]. We report a patient with recurrent pulmonary metastatic disease from a uterine leiomyosarcoma who is doing well after two lines of chemotherapy and three pulmonary metastasectomies.

## Case presentation

A 51-year-old female presented to the emergency department (ED) in December 2006 with left-sided chest pain. Past medical history was significant for a hysterectomy for a uterine fibroid one year prior. Pathology revealed a smooth muscle tumor composed of oval to spindle-shaped cells, with a plexiform pattern of growth. Although the tumor had a low mitotic rate, cells were noted to have irregular nuclei, a feature of cellular atypia.

Informed patient consent was obtained.

Chest x-ray done in the ED showed a large area of opacity in the left lung base and multiple pulmonary nodules. A follow-up CT scan of the chest showed a 10 cm mass in the left base, a left pleural effusion, and multiple bilateral pulmonary nodules suspicious for metastatic disease. A CT-guided biopsy of the mass revealed metastatic leiomyosarcoma, histologically resembling the patient’s prior uterine tumor. Immunohistochemical stains were positive for estrogen receptors (ER) and progesterone receptors (PR), and focally positive for actin, again consistent with those of the uterine tumor.

**Figure 1:**
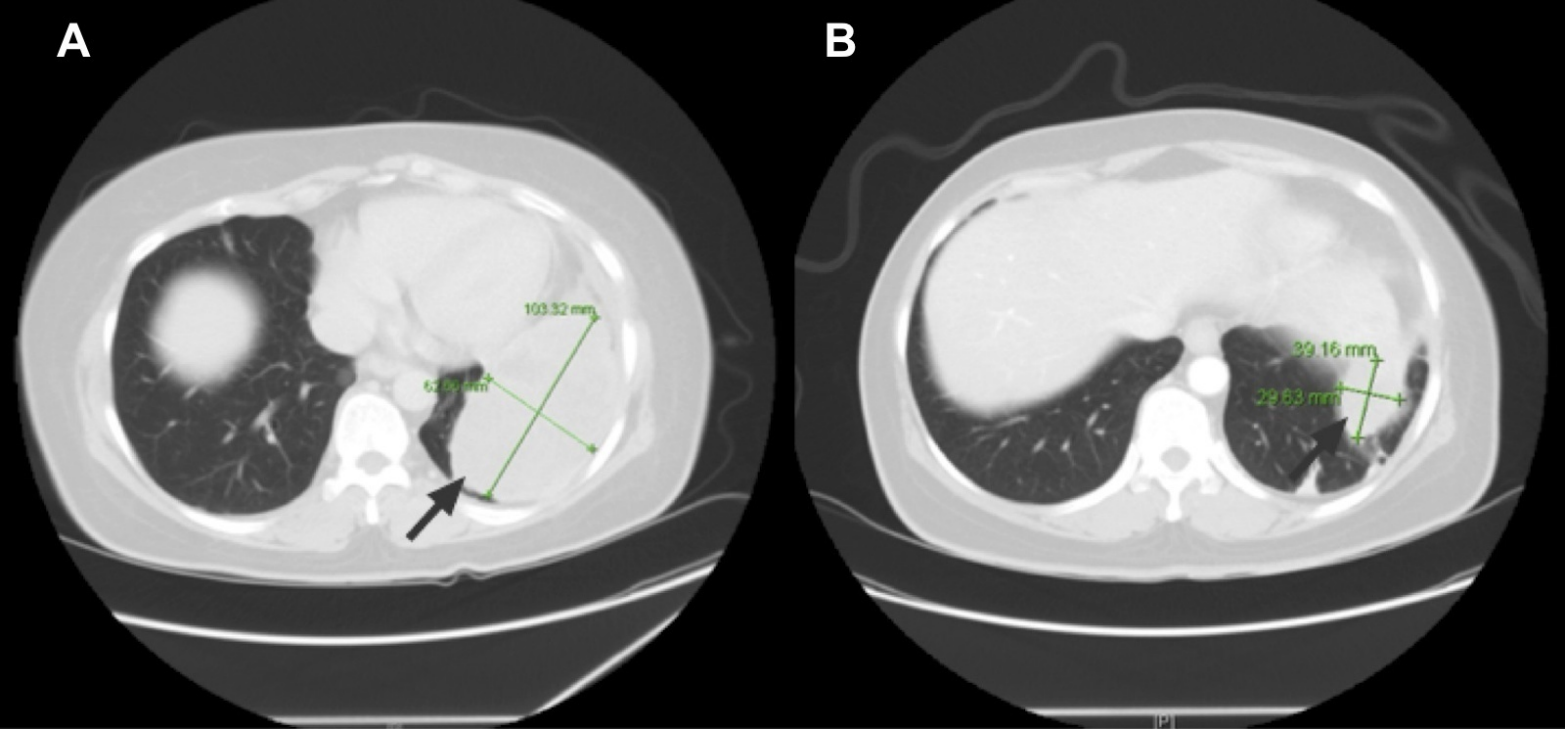
Panel A: CT chest shows a large soft tissue mass at the left lung base (arrow). Panel B: Repeat CT chest post 7 cycles of chemotherapy demonstrates a significant decrease in size of the mass (arrow).

The patient received chemotherapy with seven cycles of doxorubicin and ifosfamide, achieving a partial response (Figure 1).

She subsequently underwent metastasectomies in August and October 2007. Because the tumor was hormone receptor-positive, she was started postoperatively on Megestrol, which she took for about five years.

She remained disease-free until September 2014 when a CT scan of the chest done for a persistent cough revealed two left upper lobe nodules measuring 4.3 x 2.1 x 1.9 cm and 1.7 x 1.6 x 1.6 cm as well as a right paraesophageal mass measuring 2.3 x 1.8 x 3.5 cm. A CT-guided biopsy of one of the lesions showed metastatic leiomyosarcoma.

She then received six cycles of gemcitabine and docetaxel. Post-chemotherapy CT scan of the chest showed a decrease in the size of the nodules and the paraesophageal mass. As the patient’s tumor recurrence was again hormone receptor-positive, she was started on anastrozole, 1 mg daily.

In August 2015, the patient underwent a left upper lobe wedge resection for her two pulmonary metastases. She tolerated the procedure well. She is currently being evaluated for a metastasectomy of the right-sided lesion.

## Discussion

Leiomyosarcomas are malignant tumors originating from smooth muscle cells. They may arise from a number of primary sites, including the uterus, soft tissues, gastrointestinal tract, and blood vessel walls. The incidence rate of leiomyosarcoma is 1.23 per 100,000 person-years. The uterus is the most common site of leiomyosarcoma in women, accounting for 40% of cases [7].

On microscopy, leiomyosarcomas are characterized by cytologic atypia, tumor cell necrosis, and mitotic activity, distinguishing them from benign smooth muscle tumors [8].

In a study based on the US Surveillance, Epidemiology, and End Results data from 1988-2003, 68% of patients presented with International Federation of Gynecology and Obstetrics (FIGO) Stage I disease, 3% with Stage II disease, 7% with Stage III disease, and 22% with Stage IV disease. The five-year disease-specific overall survival was 76%, 60%, 45%, and 29% for FIGO Stages I, II, III, and IV, respectively [9].

Surgical resection with a total hysterectomy with or without bilateral salpingo-oophorectomy is the standard treatment for patients with localized disease. Currently available clinical data have not established a clear benefit for adjuvant chemotherapy or radiation therapy. Thus, observation is an acceptable management option for patients with early stage disease. For more advanced disease, adjuvant chemotherapy with or without radiation is more strongly considered, given the higher risk of recurrence in these patients. Chemotherapy is the mainstay of treatment for patients with metastatic disease [2-3].

Chemotherapeutic regimens with activity in uterine leiomyosarcoma include docetaxel/gemcitabine and doxorubicin/ifosfamide. Docetaxel/gemcitabine achieves an objective response rate (ORR) of 24% and a median progression-free survival (PFS) of 4.7 months [4]. Doxorubicin/ifosfamide has been shown to achieve an ORR of 30%, with the duration of response averaging four months [5]. Our patient received both regimens during the course of her disease. Other agents used either alone or in combination include dacarbazine, epirubicin, vinorelbine, eribulin, temozolomide, pazopanib, and trabectedin. Hormonal therapy, as our patient received, can be considered for hormone receptor-positive leiomyosarcomas [2-3].

In patients with resectable pulmonary metastases from leiomyosarcoma, aggressive and repeated metastasectomy has been shown to improve overall survival as compared to patients undergoing pulmonary metastasectomy for other types of sarcoma. In a single-institution retrospective review, overall survival was reported at 70 vs. 24 months in favor of patients with leiomyosarcoma [6].

There is a suggestion that a subset of uterine leiomyosarcomas behaves in an indolent manner. One case series of patients with hormone receptor-positive metastatic leiomyosarcoma treated with hormonal therapy reported a median PFS of 14 months. Patients with low-volume disease or moderate to strongly hormone-receptor positive disease did clinically better, with a median PFS of 20 months [10].

## Conclusions

Our patient, who remained clinically disease-free for over seven years following systemic chemotherapy and bilateral pulmonary metastasectomies, is an exceptional case among a group of patients who generally exhibit poor prognoses. Moreover, she responded to second-line chemotherapy and was considered a candidate for repeat metastasectomies. Further study on the clinical and pathologic characteristics of uterine leiomyosarcoma will help clinicians further individualize prognostication and treatment of women with this disease. 
